# Correlation between weight-bearing asymmetry and bone mineral density in patients with bilateral knee osteoarthritis

**DOI:** 10.1186/s13018-021-02252-5

**Published:** 2021-02-02

**Authors:** Kohei Nishizawa, Kengo Harato, Yutaro Morishige, Shu Kobayashi, Yasuo Niki, Takeo Nagura

**Affiliations:** Department of Orthopaedic Surgery, Keio University School of Medicine, 35 Shinanomachi, Shinjuku, Tokyo, 160-8582 Japan

**Keywords:** Knee osteoarthritis, Weight-bearing asymmetry, Gait analysis; Standing, Extension deficit, Bone mineral density

## Abstract

**Background:**

Although unloading of the joint is related to reduction of the local bone mineral density (BMD), little attention had been paid to the relationship between loading asymmetry and side-to-side difference of BMD in patients with bilateral knee osteoarthritis (OA). The aim of the present study was to evaluate and clarify the relationship between gait parameters and bone mineral density in those patients.

**Methods:**

A total of 36 knees in eighteen patients (mean age = 73.7 ± 6.3 years, mean body mass index = 26.7 ± 3.8 kg/m^2^) with bilateral medial knee OA were enrolled in the present study. All subjects performed relaxed standing and level walking at our gait laboratory after informed consent was obtained. First, ground reaction force was calculated on bilateral knees during standing. The knees in each patient were divided into higher and lower force side for the definition of dominant side limb. Second, gait parameters in each subject were obtained. To analyze the factors that affect the weight-bearing distribution in both limbs, clinical data and biomechanical parameters were compared between knees. Clinical data included radiographic OA grade, femorotibial angle, and BMD at the bilateral femoral neck.

**Results:**

Knees on higher force side were significantly more extended than on lower force side in standing (*P* = 0.012) and knee excursion during weight acceptance phase in gait was significantly larger in higher side than in lower side (*P* = 0.006), while the other parameters were not significantly different. As to the clinical data, higher force side had greater BMD, compared to lower force side. In terms of Kellgren–Lawrence scale and femorotibial angle on plain radiographs, there were no significant differences between higher and lower force side.

**Conclusions:**

Based on loading asymmetry in the present study, lower BMD was observed on Lower force side in patients with knee OA. Therefore, it is helpful for orthopedic surgeons to examine side-to-side differences of bone mineral density or extension limitation during standing for evaluation of the loading condition in patients with bilateral knee OA.

## Introduction

End-stage osteoarthritis (OA) at the knee joint is a leading cause of functional impairment and disability which is correlated with deterioration of daily activities in the elderly population. Based on previous literatures, symptomatic OA at the knee joint is seen in 14.4% of males and 28.4% of females with older than 45 years and 87% of knee OA occur bilaterally [1, 2]. Weight-bearing asymmetry in standing and walking is a well-known phenomenon in knee OA [3, 4]. It is clinically important to know the loading condition to avoid biomechanical overload and to improve treatment method for bilateral knee OA. According to previous studies, knee extension in standing seemed to be a key factor to decide side-to-side difference of loading condition in patients with knee OA [3, 5, 6].

On the other hand, several researchers investigated the relationship between bone mineral density and osteoarthritis [7–19]. For instance, some studies have indicated that OA is associated with higher bone mineral density (BMD) [19–22], and other studies have shown that high BMD decreases the risk of progression of OA [18, 23]. Thus, the relationship between OA and BMD is complicated and bone metabolism plays an important role in the pathophysiology of OA and BMD [9]. Although unloading of the joint is related to reduction of the local BMD [24], little attention had been paid to the relationship between loading asymmetry and side-to-side difference of local BMD in those with knee OA.

The purpose of the current study was to investigate weight-bearing asymmetry and to clarify the relationship between asymmetry of gait parameters and BMD. It was hypothesized that loading condition would reflect BMD at femoral neck in those with bilateral knee OA.

## Materials and methods

### Participants

A total of 36 knees in 18 patients (mean age = 73.7 ± 6.3 years, mean body mass index = 26.7 ± 3.8 kg/m^2^) participated in the present study. All patients had radiographic medial knee OA bilaterally. Radiographic OA was more severe than grade 3 severities based on the Kellgren–Lawrence scale. None of patients had any history of major trauma or injuries to lower extremities and trunk. Patients with symptomatic hip or ankle osteoarthritis or lumbar canal stenosis were excluded. Patients with rheumatoid arthritis were also excluded from the present study. An informed consent form which was approved by our Institutional Review Board was obtained in each patient.

### Gait analysis system

**Fig. 1:**
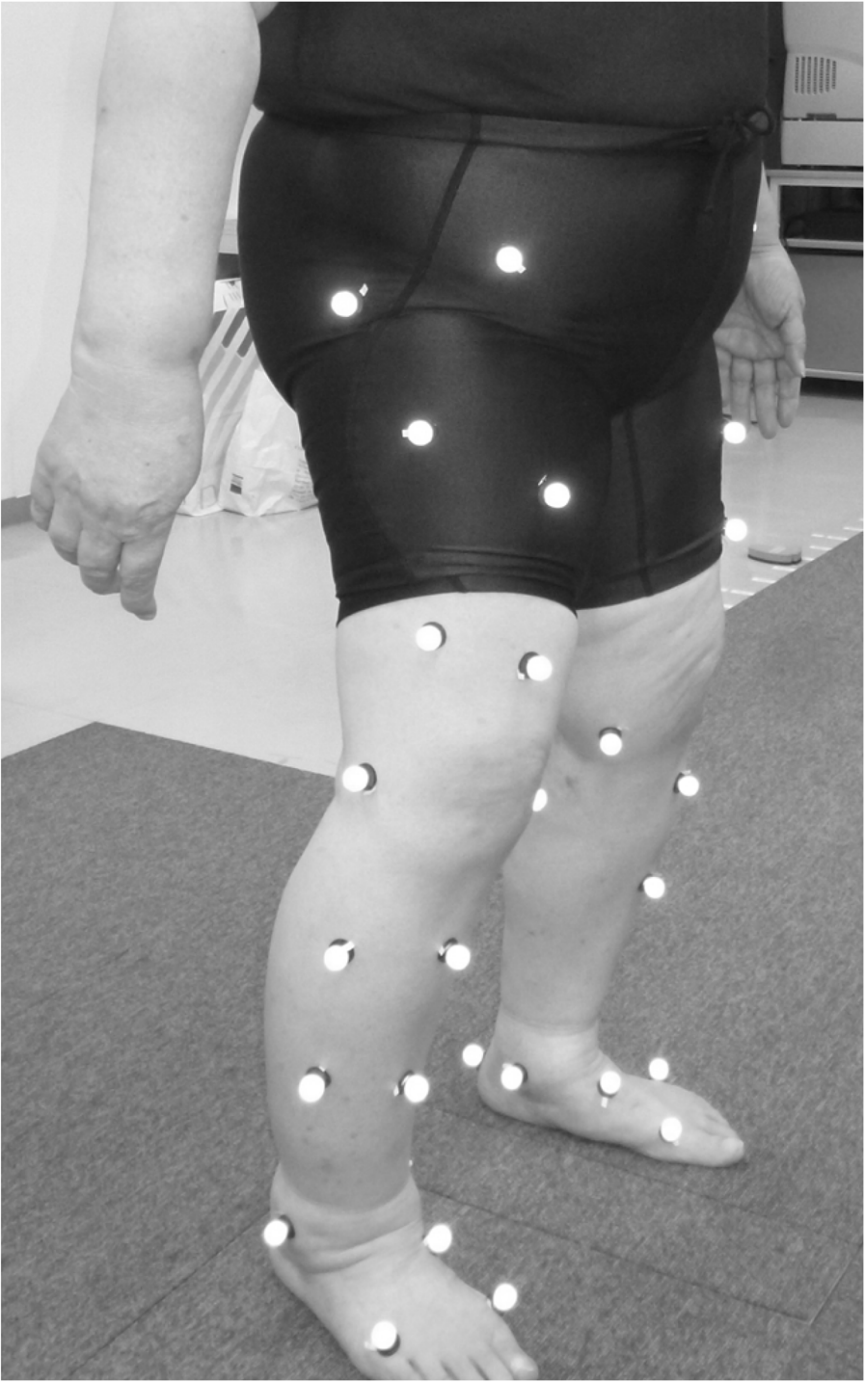
Gait analysis using a motion analysis system in patients with bilateral knee osteoarthritis

**Fig. 2:**
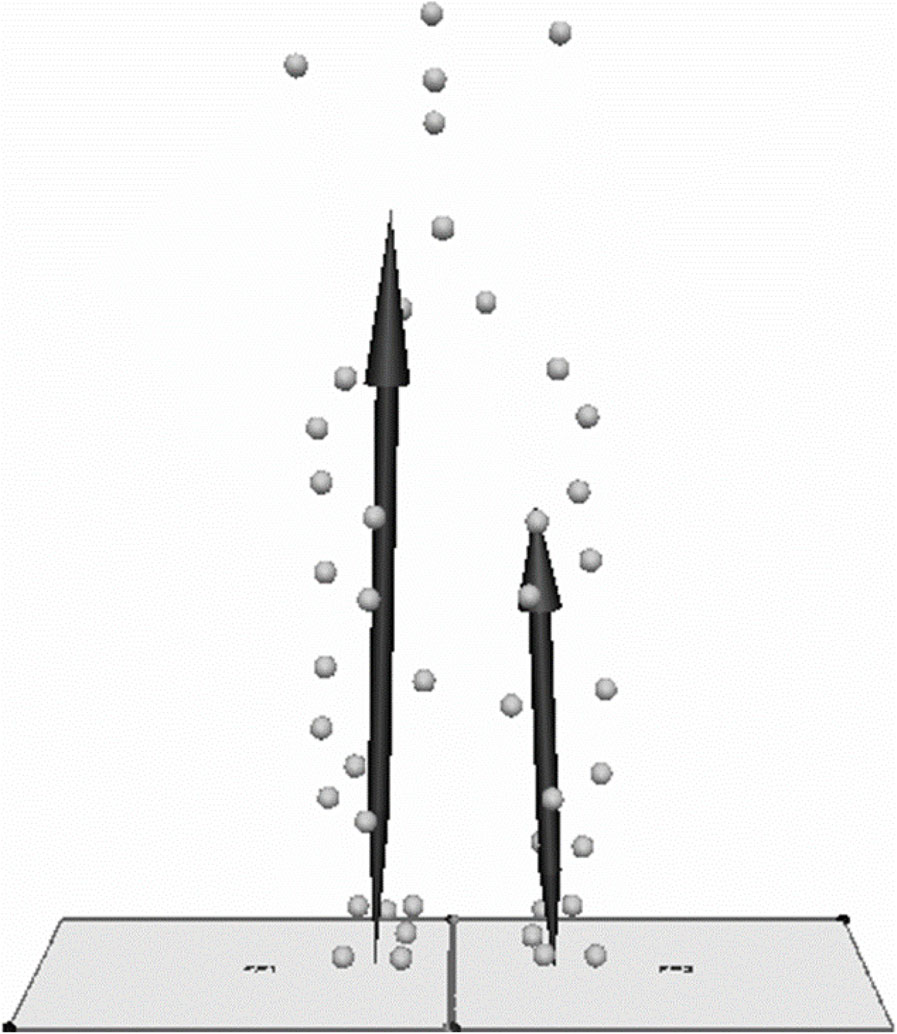
The knees in each patient were divided into higher and lower force side for the definition of dominant side limb

Gait analysis was done using motion capture system which consisted of 8 cameras (120 frames/s; Oqus, Qualisys, Sweden), 2 force plates (frequency 600 Hz; AM6110, Bertec, Columbus, OH, USA), and 46 retro-reflective markers (14 mm in diameter) (Fig. 1). All patients performed relaxed standing, placing one foot on each force plate separately, and thereafter, level walking at a preferred velocity. First, ground reaction force (GRF) was assessed on bilateral sides during standing to investigate side-to-side differences for each patient. Bilateral knees were divided into higher and lower force side based on loading condition for each patient (Fig. 2). Second, gait parameters were calculated using the average of three trials in gait. The movement of markers was recorded by Qualisys Track Manager Software (version 2.7). Visual 3D (C-motion Company, Rockville, MD, USA) was used to calculate knee biomechanics for each patient.

### Evaluations

To consider the factors affecting the loading condition on both limbs, knee data were compared. Knee data included radiographic OA grade based on the Kellgren–Lawrence scale, femorotibial angle (FTA) on plain radiograph and BMD (g/cm^2^) at the bilateral femoral neck (PRODIGY, Fuga, GE Healthcare, Buckinghamshire, UK). Since side-to-side differences of weight-bearing condition during standing would lead to the gait asymmetry as previously described in the literature [3], following parameters were also assessed; knee flexion angles (degrees) in standing and at heel strike during gait based on a kinematic waveform. Furthermore, peak values of GRF (kN/kg), peak values of net external knee adduction and flexion moment (Nm/kg), and the time from heel contact to first peak GRF (Second) during gait were also assessed.

### Statistical analysis

For the statistical analysis, regarding clinical knee data and gait parameters, two-tailed paired *t* test was performed to compare the differences between higher and lower force sides in each patient. Chi-square test was used for categorical variables. *P* values of < 0.05 were considered as significant. All statistical analyses were performed using IBM SPSS Statistics for Windows, Version 23.0 (IBM Corp., NY, USA). The sample size for the current investigation was determined to be 10 subjects in each group with 80 % power. This calculation was performed using bone mineral density, with defined significant differences of 0.02 g/cm^2^ between groups.

## Results

### Clinical data in each side

Detailed clinical data were found in Table 1. Weight-bearing asymmetry was detected between bilateral knees in all patients. In terms of Kellgren–Lawrence scale and FTA on plain radiographs, there were no significant differences between higher and lower force side. Therefore, radiographic severity was similar between both knees. On the other hand, higher force side had greater BMD, compared to lower force side.

**Table 1:** Clinical data in each side (mean ± SD)

	Higher side	Lower side	*P* value^a^
Ground reaction force in Standing (kN/kg)	0.60 ± 0.11	0.47 ± 0.13	
Kellgren–Lawrence grade (3/4)	8/10	6/12	0.73
Femorotibial angle (deg.)	184.1 ± 4.2	185.9 ± 4.5	0.12
Bone mineral density (g/cm^2^)	0.84 ± 0.13	0.81 ± 0.14	0.02

^a^ Values obtained using two-tailed paired *t* test or chi-square test

### Kinematic and kinetic data in standing and walking

**Fig. 3:**
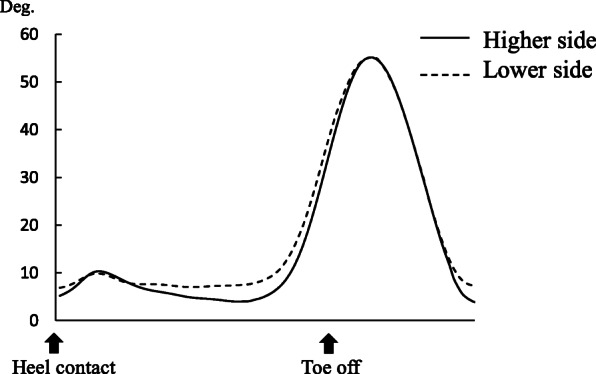
A kinematic waveform in the sagittal plane on each limb

Kinematic and kinetic data in standing and walking were found in Table 2. Knees on higher force side were significantly more extended (4.7 ± 6.9°) than those on lower force side (8.5 ± 6.6°) during relaxed standing (*P* = 0.012). A kinematic waveform in sagittal plane was presented in Fig. 3. Knee excursion on lower force side during weight acceptance phase in gait was significantly smaller than on higher force side (Table 2 and Fig. 3), while the other parameters were not significantly different.

**Table 2:** Kinematic and kinetic data in standing and walking (mean ± SD)

	Higher side	Lower side	*P* value^a^
Knee flexion angle in standing (°)	4.7 ± 6.9	8.5 ± 6.6	0.012
Knee flexion angle at heel strike in gait (°)	5.5 ± 6.1	6.7 ± 5.1	0.27
Knee excursion during weight acceptance phase in gait (°)	8.2 ± 4.1	4.5 ± 3.2	0.002
Peak knee flexion moment in gait (Nm/kg)	0.31 ± 0.24	0.33 ± 0.21	0.65
Peak knee adduction moment in gait (Nm/kg)	0.67 ± 0.19	0.70 ± 0.19	0.38
Peak ground reaction force in gait (kN/kg)	1.03 ± 0.06	1.03 ± 0.04	0.66
Time from initial contact to first peak GRF (s)	0.23 ± 0.08	0.25 ± 0.09	0.15

^a^Values obtained using two-tailed paired *t* test

## Discussion

The results of the present study supported the hypothesis that loading condition should reflect the bone mineral density at femoral neck in patients with bilateral knee OA. The most important finding of the current investigation was that larger knee extension limitation was observed in lower force side than in higher force side, as well as the side-to-side difference of BMD. Furthermore, interestingly, knee excursion on higher force side during weight acceptance phase in gait was significantly smaller than on lower force side as shown in Fig. 3. This phenomenon was well known as stiffening strategy of anterior cruciate ligament (ACL) deficient knee during gait [25]. A previous study indicated that this pattern would be related to the higher hamstring muscle activity to stabilize the ACL-deficient knee during the weight acceptance phase. However, similarly, this gait pattern was observed in posterior cruciate ligament deficient knees or knees with complete discoid lateral meniscus [26, 27]. Although the true reason of this strategy was unknown, extension limitation on lower force side was possibly related to smaller knee excursion in the sagittal plane during weight acceptance phase in the current study as a compensatory mechanics.

Linde et al. investigated the relationship between severity of knee OA and BMD using a total of 450 patients with knee OA [9]. They concluded that bone mineral density was lower with severe knee osteoarthritis. In addition, mechanical disuse could result in an imbalance of the natural resorption and formation of bone [24]. Based on loading asymmetry in the present study, lower BMD was observed on lower force side in patients with knee OA. According to previous reports, it is clinically essential to examine the ability to extend the knee during standing when considering weight-bearing condition during gait in patients with knee OA [3, 5, 6]. Similarly, extension limitation in the present study was related to weight-bearing asymmetry. On the other hand, knee kinetics, including GRF, flexion, and adduction moments, were not significantly different in the present study. This reason was that participants in the present study did not have symptomatic knees bilaterally and radiographic severity was not always same on bilateral knees in each patient [3]. From the present results, extension limitation in standing would cause mechanical disuse, and eventually lead to low BMD on the affected side. In terms of unilateral total knee arthroplasty in patients with knee OA, operated knees had dominant weight-bearing if enough extension was observed, compared to non-operated knees [5]. Therefore, to know the loading condition can be essential to decide surgery side for those patients, as mechanical disuse will improve after pain relief is obtained by successful surgery.

Several limitations should be described in the present study. First, the current investigation was not a longitudinal study. Thus, it is unknown whether high BMD will decrease the risk of progression of OA or not. Second, the present study did not evaluate bone markers such as C-terminal telopeptide of type I collagen and aminoterminal propeptide of type I collagen. Lastly, participants were selected based on the availability of BMD and gait analysis. This selection introduces a possible selection bias. Although there were several limitations, the results in the current study offer useful information when considering the relationship between BMD and the loading asymmetry in patients with bilateral end-stage knee OA. Further study will be necessary regarding the change of BMD after the appropriate treatment of knee OA.

## Conclusion

From the present study, greater BMD was observed on higher force side than on lower force side. Clinically, the orthopedic surgeons should know the side-to-side differences of BMD to evaluate the loading asymmetry in patients with knee OA. Moreover, loading asymmetry could be assessed based on the side-to-side difference of the knee extension limitation during standing.

## Data Availability

All supporting data can be provided based on request to the authors.

## Abbreviations

BMD: Bone mineral density
OA: Knee osteoarthritis
GRF: Ground reaction force
FTA: Femorotibial angle
ACL: Anterior cruciate ligament
